# Supplementary material for: The Impact of the Pandemic on Mental Health in Ethnically Diverse Mothers: Findings from the Born in Bradford, Tower Hamlets and Newham COVID-19 Research Programmes
Source: Int J Environ Res Public Health. 2022 Nov 2;19(21):14316. doi: 10.3390/ijerph192114316 (PMC9655974; doi:10.3390/ijerph192114316)
Supplement: Supplementary file 1 [file ijerph-19-14316-s001.zip › ijerph-1888332-supplementary.pdf]

Supplementary Table S1. Differences in maternal and home characteristics by levels of anxiety symptoms (N=2,807)

Characteristics	Anxiety Symptoms								Total
	None		Mild		Moderate		Severe		Total
	N	% (95% CI)	N	% (95% CI)	N	% (95% CI)	N	% (95% CI)	
**Overall**	1360	48% (47%-50%)	861	31% (29%-32%)	421	15% (14%-16%)	165	6% (5%-7%)	2,807
**Ethnicity**									
**White**									
British	533	45% (42%-47%)	445	36% (33%-39%)	172	14% (12%-16%)	67	5% (4%-7%)	1,237
Irish	15	12% (8%-20%)	45	37% (29%-46%)	59	49% (40%-58%)	<5	---	121
Any other White	87	40% (33%-46%)	69	32% (26%-38%)	50	23% (18%-29%)	13	6% (3%-10%)	219
**Black/Black British**									
Caribbean	21	46% (32%-60%)	15	33% (21%-47%)	5	11% (5%-24%)	5	11% (5%- 24%)	46
African	17	49% (33%-65%)	9	26% (14%-42%)	6	17% (8%-33%)	<5	---	35
**Asian/Asian British**									
Indian	61	65% (55%-74%)	21	22% (15%-32%)	9	10% (5%-17%)	<5	---	94
Pakistani	424	64% (61%-68%)	134	20% (17%-24%)	69	10% (8%-13%)	32	5% (3%-7%)	659
Bangladeshi	81	41% (34%- 47%)	67	34% (27%-40%)	32	16% (12%-22%)	20	10% (7%-15%)	200
Any other Asian	42	52% (41%-62%)	25	31% (22%-42%)	9	11% (6%-20%)	5	6% (3%-14%)	81
**Any other ethnic group/Mixed**	59	51% (42%-60%)	31	27% (20%-36%)	10	9% (5%-15%)	15	13% (8%-21%)	115
**Relationship status**									
Single	159	48% (43%-54%)	74	23% (18%-27%)	54	16% (13%-21%)	41	13% (9%-17%)	328
Married/civil partnership	1056	48% (46%-50%)	698	32% (30%-34%)	337	15% (14%-17%)	103	5% (4%-6%)	2,194
Not married but in a relationship	145	51% (45%-57%)	89	31% (26%-37%)	30	11% (7%-15%)	21	7% (5%-11%)	285
**Pregnancy status**									
No	1244	49% (48%-51%)	775	31% (29%-33%)	342	14% (12%-15%)	154	6% (5%-7%)	2,515
Yes	116	40% (34%-45%)	86	29% (25%-35%)	79	27% (22%-32%)	11	4% (2%-7%)	292
**Home condition – mould or damp house**									
No	1079	51% (49%-53%)	656	31% (29%-33%)	281	13% (12%-15%)	98	5% (4%-6%)	2,114
Yes	281	41% (37%-44%)	205	30% (26%-33%)	140	20% (17%-23%)	67	10% (8%-12%)	693

**Worrying about job security**									
Strongly disagree	233	61% (56%-66%)	87	23% (19%-27%)	42	11% (8%-15%)	21	5% (4%-8%)	383
Disagree	371	54% (50%-57%)	192	28% (25%-31%)	103	15% (12%-18%)	25	4% (2%-5%)	691
Neither agree or disagree	312	47% (44%-51%)	184	28% (25%-31%)	116	18% (15%-21%)	47	7% (5%-9%)	659
Agree	327	44% (41%-48%)	255	35% (31%-38%)	116	16% (13%-19%)	37	5% (4%-7%)	735
Strongly agree	117	35% (30%-40%)	143	42% (37%-48%)	44	13% (10%-17%)	35	10% (8%-14%)	339
**Food insecurity – Food didn't last**									
Never true	1172	57% (55%-59%)	611	30% (28%-32%)	200	10% (9%-11%)	78	4% (3%-5%)	2,061
Sometimes true	155	30% (26-34%)	168	33% (29%-37%)	132	26% (22%-30%)	57	11% (9%-14%)	512
Often true	33	14% (10%-19%)	82	35% (29%-41%)	89	38% (32%-44%)	30	13% (9%-18%)	234
**Food insecurity – Couldn't afford balanced meals**									
Never true	1171	57% (54%-59%)	612	30% (28%-32%)	209	10% (9%-11%)	80	4% (3%-5%)	2,072
Sometimes true	141	30% (26%-34%)	161	34% (30%-38%)	127	27% (23%-31%)	45	9% (7%-12%)	474
Often true	48	18% (14%-24%)	88	34% (28%-40%)	85	33% (27%-38%)	40	15% (11%-20%)	261
**Food insecurity – Have been hungry?**									
No	1333	54% (52%-56%)	736	30% (28%-32%)	285	12% (10%-13%)	113	5% (4%-5%)	2,467
Yes	27	8% (6%-11%)	125	37% (32%-42%)	136	40% (35%-45%)	52	15% (12%-20%)	340
**Financial insecurity – How are you getting on?**									
Living comfortably	393	66% (62%-70%)	149	25% (22%-29%)	47	8% (6%-10%)	8	1% (1%-3%)	597
Doing alright	635	54% (51%-57%)	324	28% (25%-30%)	162	14% (12%-16%)	51	4% (3%-6%)	1,172
Just about getting by	265	36% (33%-40%)	281	39% (35%-42%)	140	19% (17%-22%)	42	6% (4%-8%)	728
Finding it quite difficult	50	24% (18%-30%)	75	35% (29%-42%)	52	25% (19%-31%)	35	17% (12%-22%)	212
Finding it very difficult	17	17% (11%-26%)	32	33% (24%-43%)	20	20% (14%-30%)	29	30% (21%-39%)	98
**Quality of relationship with partner**									
NA- Single	159	48% (43%-54%)	74	23% (18%-27%)	54	16% (13%-21%)	41	13% (9%-17%)	328
Excellent	636	63% (60%-66%)	258	26% (23%-29%)	76	8% (6%-9%)	32	3% (2%-4%)	1,002
Good	475	45% (42%-48%)	373	35% (33%-38%)	161	15% (13%-18%)	42	4% (3%-5%)	1,051
Average	79	23% (19%-27%)	134	39% (34%-44%)	108	31% (26%-36%)	27	8% (5%-11%)	348
Poor	8	16% (8%-29%)	16	32% (21%-46%)	15	30% (19%-44%)	11	22% (13%-36%)	50
Very poor	<5	---	6	21% (10%-40%)	7	25% (12%-44%)	12	43% (26%-61%)	28
**Social support – No. of people you can count on**									
0-2: Low	229	34% (30%-37%)	245	36% (32%-40%)	141	21% (18%-24%)	68	10% (8%-12%)	683
3-6: Medium	644	46% (44%-49%)	460	33% (31%-35%)	226	16% (14%-18%)	67	5% (4%-6%)	1,397
7 and more: High	487	67% (63%-70%)	156	21% (19%-25%)	54	7% (6%-10%)	30	4% (3%-6%)	727

**Social support – No. of people you can count on living locally**									
0-2: Low	552	40% (37%-42%)	513	37% (35%-40%)	218	16% (14%-18%)	103	7% (6%-9%)	1,386
3-6: Medium	568	53% (50%-56%)	282	26% (24%-29%)	174	16% (14%-19%)	45	4% (3%-6%)	1,069
7 and more: High	240	68% (63%-73%)	66	19% (15%-23%)	29	8% (6%-12%)	17	5% (3%-8%)	352
**Loneliness**									
None/almost none of the time	941	72% (69%-74%)	271	21% (19%-23%)	76	6% (5%-7%)	25	2% (1%-3%)	1,313
Some of the time	373	37% (35%-41%)	398	40% (37%-43%)	174	17% (15%-20%)	50	5% (4%-7%)	995
Most of the time	39	10% (8%-14%)	165	43% (38%-48%)	127	33% (29%-38%)	53	14% (11%-18%)	384
All/almost all of the time	7	6% (3%-12%)	27	23% (17%-32%)	44	38% (30%-47%)	37	32% (24%-41%)	115

Supplementary Table S2. Predictive probabilities (as percentages) and the corresponding 95% CIs of clinically important depressive by ethnicity for the effect of financial insecurity during the pandemic (n=2,807)

**Model (a): Ethnicity only**	**Est.**	**95% CI: Low**	**95% CI: High**
White: British	27.3%	24.8%	29.8%
White: Other	49.7%	44.4%	55.0%
Black: Caribbean/African	29.6%	19.7%	39.6%
Asian: Indian	13.8%	6.9%	20.8%
Asian: Pakistani	17.8%	14.8%	20.7%
Asian: Bangladeshi	34.5%	27.9%	41.1%
Other	24.0%	18.0%	30.0%
**Model (b): Ethnicity adjusted for financial insecurity only**			
White: British	28.8%	26.2%	31.3%
White: Other	47.7%	42.5%	52.9%
Black: Caribbean/African	27.0%	17.8%	36.2%
Asian: Indian	15.3%	7.8%	22.7%
Asian: Pakistani	17.7%	14.8%	20.5%
Asian: Bangladeshi	29.5%	23.5%	35.5%
Other	23.5%	17.7%	29.3%
**Model (c): Ethnicity adjusted for location of residency & ethnicity*financial insecurity interaction**			
White: British	27.8%	25.3%	30.2%
White: Other	39.0%	33.9%	44.0%
Black: Caribbean/African	25.5%	16.6%	34.4%
Asian: Indian	14.3%	7.0%	21.5%
Asian: Pakistani	25.1%	21.1%	29.0%
Asian: Bangladeshi	24.0%	18.3%	29.7%
Other	20.6%	15.5%	25.6%
**Model (d): Ethnicity adjusted for location, ethnicity*financial insecurity interaction, and ethnicity*Social support**			
White: British	28.0%	25.4%	30.3%
White: Other	36.3%	31.7%	40.9%
Black: Caribbean/African	24.1%	15.0%	33.2%
Asian: Indian	13.4%	6.6%	20.3%
Asian: Pakistani	24.0%	20.2%	27.9%
Asian: Bangladeshi	25.3%	19.4%	31.1%
Other	20.8%	15.7%	26.0%

Abbreviations: CI, confidence interval; Est, estimate

Supplementary Table S3. Predictive probabilities (as percentages) and the corresponding 95% CIs of clinically important depressive by ethnicity for the effect of loneliness during the pandemic (n=2,807)

**Model (a): Ethnicity adjusted for loneliness only**	**Est.**	**95% CI: Low**	**95% CI: High**
White: British	26.6%	24.4%	28.8%
White: Other	36.8%	32.3%	41.4%
Black: Caribbean/African	30.2%	21.0%	39.4%
Asian: Indian	18.6%	10.6%	26.6%
Asian: Pakistani	23.6%	20.4%	26.9%
Asian: Bangladeshi	32.1%	26.5%	37.8%
Other	26.2%	20.6%	31.8%
**Model (b): Ethnicity adjusted for location & ethnicity*loneliness interaction**			
White: British	26.6%	24.3%	28.8%
White: Other	30.8%	26.8%	34.7%
Black: Caribbean/African	31.6%	22.0%	41.2%
Asian: Indian	19.2%	10.5%	27.9%
Asian: Pakistani	29.1%	25.5%	32.6%
Asian: Bangladeshi	28.9%	23.9%	34.0%
Black/ Mixed/ Other	25.4%	22.0%	31.2%
**Model (c): Ethnicity adjusted for location, ethnicity*loneliness interaction, and ethnicity*Social support**			
White: British	27.2%	24.9%	29.4%
White: Other	29.6%	25.7%	33.6%
Black: Caribbean/African	30.6%	20.5%	40.8%
Asian: Indian	17.2%	9.1%	25.4%
Asian: Pakistani	28.1%	24.5%	31.7%
Asian: Bangladeshi	29.7%	24.6%	34.8%
Other	25.5%	20.1%	30.9%

Abbreviations: CI, confidence interval; Est, estimate

Supplementary Table S4. Associations between ethnicity and clinically important anxiety symptoms (n=2,807)

	Univariate	Multivariate			
	**Unadjusted**	**Adjusted for location of residency only**	**Adjusted for loneliness only**	**Adjusted for social support only**	**Adjusted for financial insecurity only**
	**OR (95% CI)**	**OR (95% CI)**	**OR (95% CI)**	**OR (95% CI)**	**OR (95% CI)**
**Ethnicity**					
White other	2.40 (1.84-3.11)	1.82 (1.39-2.39)	1.63 (1.22-2.17)	2.08 (1.60-2.72)	2.14 (1.64-2.80)
Black: Caribbean/African	1.28 (0.75-2.18)	1.25 (0.73-2.15)	1.42 (0.78-2.56)	1.15 (0.67-1.98)	1.06 (0.62-1.83)
Asian: Indian	0.61 (0.33-1.14)	0.63 (0.34-1.18)	0.86 (0.44-1.67)	0.62 (0.33-1.16)	0.63 (0.33-1.18)
Asian: Pakistani	0.75 (0.58-0.97)	1.16 (0.87-1.54)	1.12 (0.85-1.47)	0.78 (0.60-1.01)	0.69 (0.54-0.90)
Asian: Bangladeshi	1.47 (1.04-2.07)	1.16 (0.81-1.65)	1.45 (1.00-2.12)	1.41 (0.99-2.01)	1.11 (0.78-1.56)
Other ethnicities	1.04 (0.71-1.51)	0.92 (0.63-1.35)	1.24 (0.83-1.86)	0.98 (0.67-1.44)	0.94 (0.64-1.39)
White British	1.00	1.00	1.00	1.00	1.00
**Location of residency**					
London	2.53 (2.09-3.06)	2.34 (1.86-2.96)			
Bradford	1.00	1.00			
**Loneliness**					
Some of the time	3.49 (2.71-4.48)		3.37 (2.61-4.36)		
All of the time	13.16 (10.06-17.21)		12.29 (9.31-16.22)		
None of the time	1.00		1.00		
**Social Support**					
Medium (3-6)	2.03 (1.56-2.64)			1.88 (1.44-2.44)	
Low (0-2)	3.37 (2.55-4.46)			2.97 (2.23-3.94)	
High (+7)	1.00			1.00	
**Financial insecurity**					
Insecure	2.47 (2.05-2.98)				2.39 (1.98-2.89)
Secure	1.00				1.00

Abbreviations: CI, confidence interval; OR, odds ratio

Supplementary Table S5. Predictive probabilities (as percentages) and the corresponding 95% CIs of clinically important anxiety by ethnicity for the effect of financial insecurity during the pandemic (n=2,807)

**Model (a): Ethnicity only**	**Est.**	**95% CI: Low**	**95% CI: High**
White: British	19.3%	17.1%	21.5%
White: Other	36.5%	31.3%	41.6%
Black: Caribbean/African	23.4%	14.2%	32.7%
Asian: Indian	12.8%	6.0%	19.5%
Asian: Pakistani	15.3%	12.6%	18.1%
Asian: Bangladeshi	26.0%	19.9%	32.1%
Other	19.9%	14.3%	25.5%
**Model (b): Ethnicity adjusted for financial insecurity only**			
White: British	20.4%	18.1%	22.6%
White: Other	34.7%	29.8%	39.7%
Black: Caribbean/African	21.4%	12.9%	29.9%
Asian: Indian	14.0%	6.8%	21.2%
Asian: Pakistani	15.2%	12.5%	17.9%
Asian: Bangladeshi	22.1%	16.7%	27.5%
Other	19.5%	14.1%	24.9%
**Model (c): Ethnicity adjusted for location of residency & ethnicity*financial insecurity**			
White: British	19.9%	17.7%	22.1%
White: Other	28.9%	24.2%	33.6%
Black: Caribbean/African	22.6%	13.4%	31.8%
Asian: Indian	14.0%	6.6%	21.3%
Asian: Pakistani	20.5%	16.7%	24.3%
Asian: Bangladeshi	18.1%	13.0%	23.2%
Other	17.4%	12.5%	22.3%
**Model (d): Ethnicity adjusted for location, ethnicity*financial insecurity interaction, and ethnicity*Social support**			
White: British	20.1%	17.8%	22.3%
White: Other	27.3%	22.8%	31.7%
Black: Caribbean/African	21.4%	11.9%	30.9%
Asian: Indian	13.3%	6.3%	20.2%
Asian: Pakistani	20.0%	16.2%	23.7%
Asian: Bangladeshi	18.7%	13.4%	23.9%
Other	17.3%	12.5%	22.2%

Abbreviations: CI, confidence interval; Est, estimate

Supplementary Table S6. Predictive probabilities (as percentages) and the corresponding 95% CIs of clinically important anxiety by ethnicity for the effect of loneliness during the pandemic (n=2,807)

**Model (a): Ethnicity adjusted for loneliness only**	**Est.**	**95% CI: Low**	**95% CI: High**
White: British	18.8%	16.8%	20.8%
White: Other	25.9%	22.0%	29.9%
Black: Caribbean/African	23.7%	15.0%	32.4%
Asian: Indian	16.9%	9.0%	24.8%
Asian: Pakistani	20.0%	17.1%	23.5%
Asian: Bangladeshi	24.1%	18.9%	29.4%
Black/ Mixed/ Other	21.8%	16.3%	27.2%
**Model (b): Ethnicity adjusted for location & ethnicity*loneliness interaction**			
White: British	18.8%	16.8%	20.9%
White: Other	23.0%	19.2%	26.9%
Black: Caribbean/African	25.1%	16.2%	34.0%
Asian: Indian	19.2%	11.1%	27.3%
Asian: Pakistani	24.1%	20.4%	27.9%
Asian: Bangladeshi	22.5%	17.5%	27.5%
Other	21.5%	16.3%	26.7%
**Model (c): Ethnicity adjusted for location, ethnicity*loneliness interaction, and ethnicity*Social support**			
White: British	19.3%	17.2%	21.4%
White: Other	22.4%	18.5%	26.3%
Black: Caribbean/African	25.5%	15.9%	35.1%
Asian: Indian	17.8%	9.2%	26.4%
Asian: Pakistani	23.5%	19.8%	27.3%
Asian: Bangladeshi	22.7%	17.6%	27.8%
Other	20.9%	15.5%	26.3%

Abbreviations: CI, confidence interval; Est, estimate
